# Glutamine Metabolism Regulators Associated with Cancer Development and the Tumor Microenvironment: A Pan-Cancer Multi-Omics Analysis

**DOI:** 10.3390/genes12091305

**Published:** 2021-08-25

**Authors:** Jingwen Zou, Kunpeng Du, Shaohua Li, Lianghe Lu, Jie Mei, Wenping Lin, Min Deng, Wei Wei, Rongping Guo

**Affiliations:** 1 Department of Liver Surgery, Sun Yat-sen University Cancer Center, Guangzhou 510060, China; zoujw@sysucc.org.cn (J.Z.); lishaoh@sysucc.org.cn (S.L.); lulh@sysucc.org.cn (L.L.); meijie@sysucc.org.cn (J.M.); linwp@sysucc.org.cn (W.L.); dengmin@sysucc.org.cn (M.D.); weiwei@sysucc.org.cn (W.W.); 2 State Key Laboratory of Oncology in South China, Collaborative Innovation Center for Cancer Medicine, Guangzhou 510060, China; 3 Department of Radiation Oncology, Affiliated Cancer Hospital & Institute of Guangzhou Medical University, Guangzhou 510095, China; dukunpeng@gzhmu.edu.cn

**Keywords:** glutamine, metabolism, immune, PD-L1, pan-cancer, multi-omics

## Abstract

Background: In recent years, metabolic reprogramming has been identified as a hallmark of cancer. Accumulating evidence suggests that glutamine metabolism plays a crucial role in oncogenesis and the tumor microenvironment. In this study, we aimed to perform a systematic and comprehensive analysis of six key metabolic node genes involved in the dynamic regulation of glutamine metabolism (referred to as GLNM regulators) across 33 types of cancer. Methods: We analyzed the gene expression, epigenetic regulation, and genomic alterations of six key GLNM regulators, including *SLC1A5*, *SLC7A5*, *SLC3A2*, *SLC7A11*, *GLS*, and *GLS2*, in pan-cancer using several open-source platforms and databases. Additionally, we investigated the impacts of these gene expression changes on clinical outcomes, drug sensitivity, and the tumor microenvironment. We also attempted to investigate the upstream microRNA–mRNA molecular networks and the downstream signaling pathways involved in order to uncover the potential molecular mechanisms behind metabolic reprogramming. Results: We found that the expression levels of GLNM regulators varied across cancer types and were related to several genomic and immunological characteristics. While the immune scores were generally lower in the tumors with higher gene expression, the types of immune cell infiltration showed significantly different correlations among cancer types, dividing them into two clusters. Furthermore, we showed that elevated GLNM regulators expression was associated with poor overall survival in the majority of cancer types. Lastly, the expression of GLNM regulators was significantly associated with PD-L1 expression and drug sensitivity. Conclusions: The elevated expression of GLNM regulators was associated with poorer cancer prognoses and a cold tumor microenvironment, providing novel insights into cancer treatment and possibly offering alternative options for the treatment of clinically refractory cancers.

## 1. Introduction

Metabolic reprogramming has been recognized as a hallmark of cancer in recent years [1]. The altered cell metabolism allows tumors to fulfill their increased energetic and biosynthetic requirements. Glutamine metabolism has been shown to be involved in the maintenance and survival of tumor cells and is associated with the control of oxidative stress through glutathione synthesis [2]. Recent studies have shown that the activation of tumor-specific signaling pathways, such as the upregulation of the oncogene *Myc*, can regulate glutamine uptake and metabolism through glutaminolysis, providing an alternative energy source for cancer cells [3]. Glutamine and its metabolites are involved in several key cellular processes, including the tricarboxylic acid (TCA) cycle, redox balance, and mTOR activation, as well as the biosynthesis of nucleotides, amino acids, fatty acids, and amino sugars [4,5]. Increasing evidence suggests that metabolic reprogramming plays a vital role not only in tumorigenesis and tumor progression but also in regulating the immune microenvironment. Additionally, blocking the glutamine metabolism in tumor cells, which results in an elevated level of amino acids in the tumor microenvironment (TME), can enhance the antitumor effects of immune cells; therefore, an in-depth exploration of the genetic alteration of glutamine metabolism in tumor development and immune escape may provide an important theoretical basis for developing novel cancer treatments.

Glutamine uptake and the rate of glutaminolysis are known to be associated with tumor growth [6]. Two key transporters for glutamine uptake into cells are solute carrier family 1 member 5 (*SLC1A5*) and solute carrier family 7 member 5 (*SLC7A5*). *SLC1A5* mediates the sodium (Na+)-coupled influx of glutamine, whereas *SLC7A5* maintains the efflux of glutamine alongside the influx of leucine, an essential amino acid and effective activator of mTORC1 [7,8]. *SLC7A5* requires covalent binding with the solute carrier family 3 member 2 (*SLC3A2*) heavy chain in order to be functionally expressed in the plasma membrane [9]. Solute carrier family 7 member 11 (*SLC7A11*), also called xCT, is responsible for the counter-transport of glutamate and cysteine. This exchange is favorable for cancer cells because cysteine is a major component of the antioxidant glutathione, which in turn is an antagonist of reactive oxygen species (ROS) [10,11,12]. Mitochondrial glutaminase (GLS) is the primary enzyme of glutaminolysis, serving as a gatekeeper. Two isozymes of GLS have been identified: a kidney-type enzyme (GLS) and a liver-type enzyme (GLS2) [13]. Abnormal expression patterns for the above glutamine metabolism (GLNM) regulators have been observed in some cancers and are correlated with patient survival. To date, previous studies have focused on either a single cancer type or a single gene [8,14]. There has been no comprehensive analysis of the genomic changes of the GLNM regulators and their effects on the TME and patient prognosis across different cancer types.

Drug resistance remains a major clinical issue that impairs the efficacy of cancer therapy. Currently, several mechanisms of drug resistance have been identified, including changes in drug transport, DNA repair, apoptosis, autophagy, pyroptosis, and redox balance, which was mainly mediated by reduced glutathione [15]; thus, glutamine metabolism may play an important role in drug response by maintaining redox balance. It has been reported that the upregulation of *SLC7A11* has been identified as a mechanism of cisplatin resistance in ovarian and bladder cancer, as well as gemcitabine resistance in pancreatic cancer [16,17]. This affords the opportunity to provide a fruitful theoretical basis for the implementation of individualized therapy based on the gene expression profile; therefore, comprehensive analysis of the relationships between GLNM regulators and drug sensitivity are indispensable.

In this study, we systematically analyzed the gene expression, epigenetic regulation, and genomic alterations of six key GLNM regulators (including *SLC1A5*, *SLC7A5*, *SLC3A2*, *SLC7A11*, *GLS*, and *GLS2*) across 33 different cancer types. Additionally, the impacts of these gene expression changes on clinical outcomes, drug sensitivity, and immune cell infiltration were investigated. We also explored the upstream microRNA–mRNA molecular networks and the possible downstream signaling pathways, which might be mediated through these GLNM regulators.

## 2. Materials and Methods

### 2.1. Data Acquisition

All the datasets analyzed in the present study are publicly available. The RNA-seq, single-nucleotide variation (SNV), copy number variation (CNV), methylation, and clinical data for adrenocortical carcinoma (ACC), bladder urothelial carcinoma (BLCA), breast cancer (BRCA), cervical squamous cell carcinoma and endocervical adenocarcinoma (CESC), cholangiocarcinoma (CHOL), colon adenocarcinoma (COAD), lymphoid neoplasm diffuse large b-cell lymphoma (DLBC), esophageal carcinoma (ESCA), glioblastoma multiforme (GBM), head and neck squamous carcinoma (HNSC), kidney chromophobe (KICH), kidney renal clear cell carcinoma (KIRC), kidney renal papillary cell carcinoma (KIRP), acute myeloid leukemia (LAML), brain lower-grade glioma (LGG), liver hepatocellular carcinoma (LIHC), lung adenocarcinoma (LUAD), lung squamous cell carcinoma (LUSC), mesothelioma (MESO), ovarian serous cystadenocarcinoma (OV), pancreatic adenocarcinoma (PAAD), pheochromocytoma and paraganglioma (PCPG), prostate adenocarcinoma (PRAD), rectum adenocarcinoma (READ), sarcoma (SARC), skin cutaneous melanoma (SKCM), stomach adenocarcinoma (STAD), testicular germ cell tumors (TGCT), thyroid carcinoma (THCA), thymoma (THYM), uterine corpus endometrial carcinoma (UCEC), uterine carcinosarcoma (UCS), and uveal melanoma (UVM) were obtained from the Cancer Genome Atlas (TCGA) data portal (https://gdc.cancer.gov/, accessed on 1 May 2021). These datasets included 10,995 level 3 RNA-seq samples, while clinical data were available for analysis for 11,160 samples. The Genotype-Tissue Expression (GTEx) dataset (V7.0) consists of 11,688 samples and contains 56,202 genes representing the expression profiles of 30 different organs (53 tissues), including adipose tissue, adrenal gland, bladder, blood, blood vessel, brain, breast, cervix uteri, colon, esophagus, fallopian tube, heart, kidney, liver, lung, muscle, nerve, ovary, pancreas, pituitary, prostate, salivary gland, skin, small intestine, spleen, stomach, testis, thyroid, uterus, and vagina profiles.

### 2.2. Genome-Wide Analysis

A genome-wide analysis of the GLNM genes was performed using the GSCALite platform [18]. The expression profiles of the GLNM genes in selected normal GTEx tissues were displayed in the form of a heatmap. RSEM-normalized RNA-seq data were used for differentially expressed gene (DEG) analysis. The genes with fold change values (FC) > 2 and false discovery rates (FDR) < 0.05 were identified as DEGs. The SNV frequency and variant types of the six GLNM genes were visualized in a heatmap and a waterfall plot. The CNV statistics were based on processed data from GISTIC2.0 [19], while the correlation between the CNV and mRNA expression was determined from raw data. The percentages of heterozygous and homozygous CNVs for each cancer type were compiled into a pie chat and the Pearson coefficient of the correlation between gene expression and the CNV was calculated. The methylation analysis explored the differential methylation levels and the correlation between methylation and gene expression.

### 2.3. Survival Analysis

RSEM-normalized expression values and the methylation levels of genes were used to divide the cancer samples into high and low groups based on the medians. Then, an R package, “survival”, was performed to evaluate the survival differences between the two groups. In addition, the integrative effect of the six GLNM genes on survival was investigated in the form of gene signatures using the Gene Expression Profiling Interactive Analysis (GEPIA) database [20]. The Cox proportional hazards model was used to calculate the hazards of the high-expression group [21]. The survival curves were plotted using the Kaplan–Meier method and compared between groups using log-rank tests [22]. A *p*-value less than 0.05 was considered to indicate a significant difference.

### 2.4. Analysis of Immune Cell Infiltration

We used immuneeconv, an R software package, to calculate the immune score, stroma score, and microenvironment score for each of the GLNM regulators based on xCell algorithms [23]. GEPIA2 was used to evaluate the association between PD-L1 and the gene set expression level. The association between tumor-infiltrating immune cells (TIICs) across 33 cancer types and the gene set expression level was estimated via ImmuCellAI [24]. The Spearman correlation test was applied to analyze the correlation between the expression of GLNM regulators and the abundance of different immune cells.

### 2.5. Drug Sensitivity Analysis

The gene expression data and IC50 values for different drugs for 1018 cancer cell lines in the GDSC database were integrated for investigation. The area under the dose–response curve (AUC) values for the drug and gene expression profiles were downloaded to analyze the correlation of gene expression and drug sensitivity. The Pearson correlation coefficients of annotated drug–target pairs were used for comparisons, with the same number of correlation pairs generated by randomly sampling the correlations.

### 2.6. Analysis of Molecular Mechanisms

The upstream microRNA–mRNA molecular networks and the downstream pathways activity were explored. The miRNA transcript expression data were derived from TCGA, including 9105 samples and 33 cancer types. The microRNA regulation data were obtained from databases, including experimental validation (papers, TarBase, miRTarBase, and mir2disease), as well as TargetScan and miRNADA prediction. The downstream signaling pathways activity were analyzed based on reverse-phase protein array (RPPA) data from the TCPA database. The pathway activity score (PAS) was the sum of the relative protein levels of all positive regulatory components minus the relative protein levels of the negative regulatory components in a particular pathway [25]. The cancer samples were stratified into two groups based on the median gene expression, and Student’s *t*-test was used to determine the difference in the PAS between the two groups. When a gene in the high expression group has a higher PAS than the low expression group, the gene may have an activating effect on a certain pathway or may otherwise have an inhibitory effect on the pathway [26].

### 2.7. Statistical Analysis

Student’s *t*-test was performed to compare the GLNM gene expressions differences, as well as the methylation differences between tumors and corresponding normal tissue. The percentage of the SNV in the coding region of each gene was calculated with the following formula: [number of mutated sample]/[number of cancer sample]. The log-rank test was used to compare the survival curves. The correlations were evaluated by Pearson or Spearman tests. A *p*-value less than 0.05 was considered statistically significant.

## 3. Results

### 3.1. Aberrant Expression of GLNM Regulators in a Variety of Cancers

**Figure 1:**
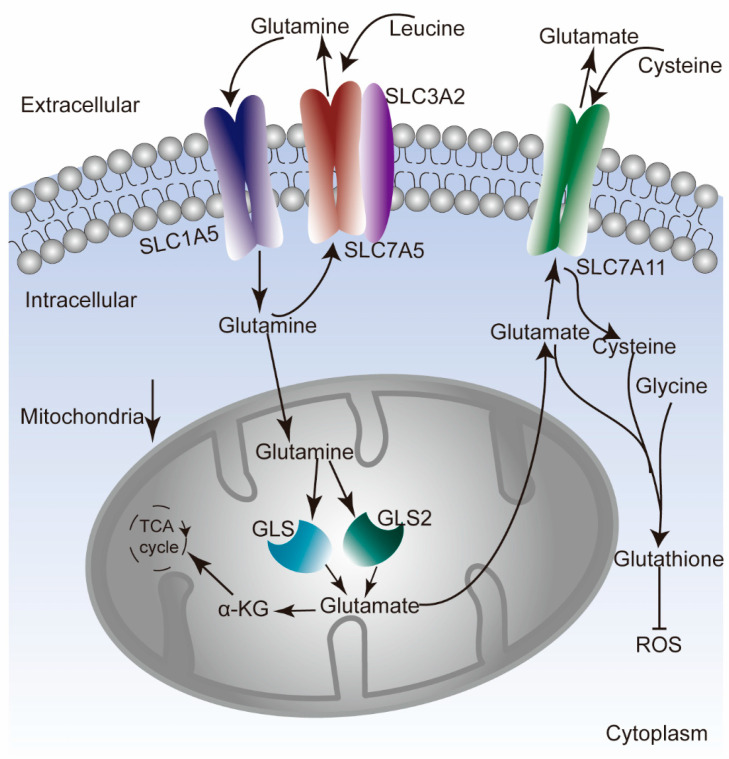
The main pathways of glutamine uptake and catabolism.

**Figure 2:**
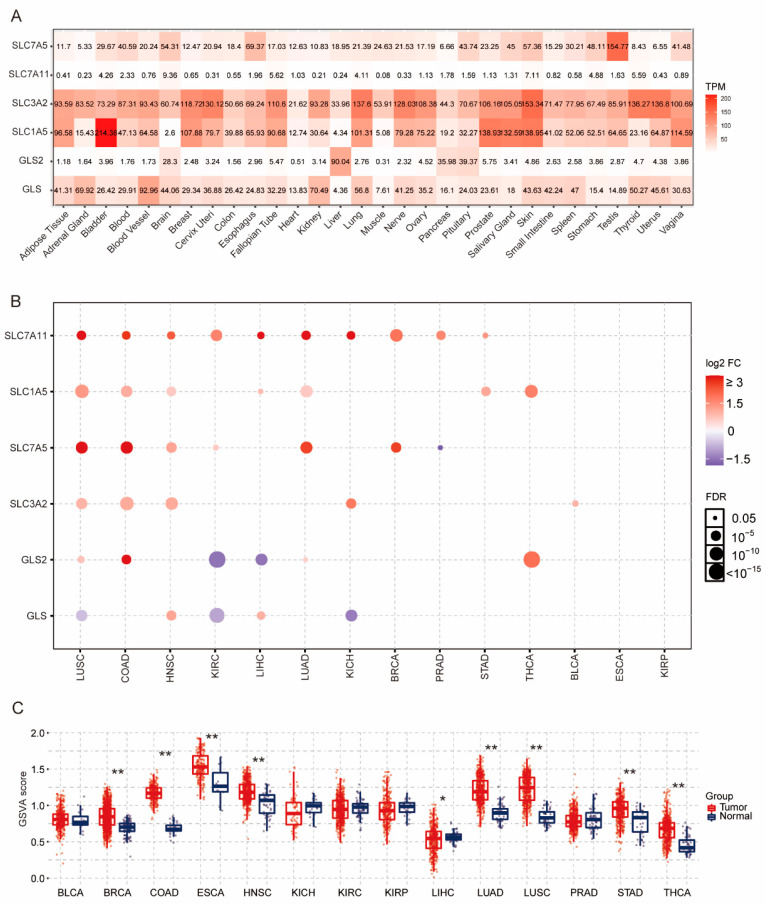
An mRNA expression comparison of glutamine metabolism (GLNM) regulators: (**A**) expression profiles of six GLNM regulators in 30 normal tissues; (**B**) differential expression levels of GLNM regulators between 14 paired normal and tumor tissues. The colors from purple to red represent the fold changes between tumor and normal tissues. Red dots represent higher expression levels of genes in tumor tissues than in normal tissues, while blue dots indicate the opposite expression pattern. The size of a dot indicates the FDR significance, while the value of each dot size is presented on the right side; (**C**) Gene Set Variation Analysis (GSVA) score based on GLNM regulators between 14 paired normal and tumor tissues. * *p* < 0.05 and ** *p* < 0.01.

Based on the published data, a total of six key GLNM regulators, including four transporters (*SLC1A5*, *SLC7A5*, *SLC3A2*, and *SLC7A11*) and two gatekeeper enzymes (*GLS* and *GLS2*), were employed in the current study (Figure 1). The expression profiles of normal tissues were assessed based on the GTEx dataset, as shown in Figure 2A. *SLC3A2* and *SLC1A5* were highly expressed in a variety of tissues, while *SLC7A11* displayed lower expression. The expression levels of *SLC7A5* in the testis, *GLS2* in the liver, and *GLS* in blood vessel were relatively high. The differential expression analysis was based on paired samples for each cancer type. Across the 33 cancer types, only THCA, KIRP, BLCA, LIHC, HNSC, BRCA, LUAD, PRAD, ESCA, KICH, LUSC, KIRC, STAD, and COAD had paired samples (Figure 2B). Upregulated expression of *SLC7A11* (10/14), *SLC1A5* (7/14), *SLC7A5* (6/14), and *SLC3A2* (5/14) was observed in most of the tumor tissues; however, *GLS* in LUSC, KIRC, and KICH and *GLS2* in KIRC and LIHC, were significantly downregulated. Next, a gene set variation analysis (GSVA) was performed to calculate a GSVA score to represent the integrated level of the expression of the gene set, which was positively correlated with the expression of the gene set; therefore, if the GSVA score in the tumor group was higher than that in adjacent group, this indicated that the overall expression of the gene set in the tumor group was higher. In the present study, we observed that the GSVA scores were significantly higher in BRCA, COAD, ESCA, HNSC, LIHC, LUAD, LUSC, STAD, and THCA than in the adjacent normal tissues (Figure 2C). This revealed that the aberrant expression of the GLNM regulators might be involved in tumorigenesis in multiple cancer types.

### 3.2. Genetic Alterations of GLNM Regulators in Various Cancers

**Figure 3:**
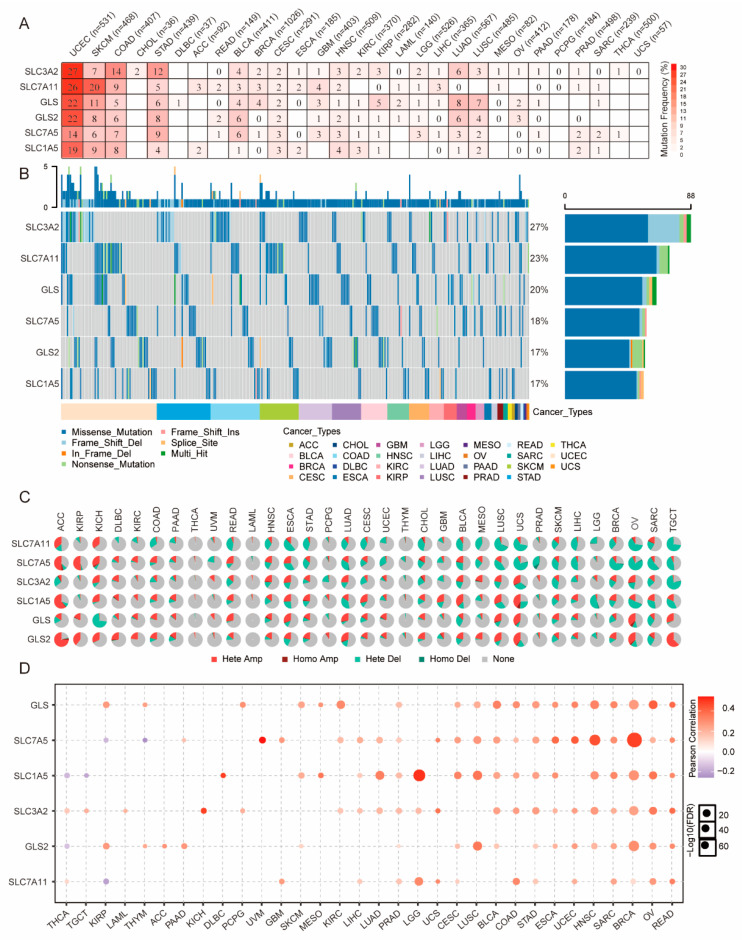
Associations between glutamine metabolism (GLNM) regulators, genomic features, and expression: (**A**) heatmap showing the single-nucleotide variation (SNV) frequencies of six GLNM regulators across different cancer types; (**B**) waterfall plot showing the mutation distribution and classification of SNV types; (**C**) copy number variation (CNV) pie plot showing the proportions of different types of CNVs in each gene across different cancer types; (**D**) correlation between CNV and mRNA expression. Blue dots represent negative correlations and red dots represent positive correlations. The sizes of dots represent significance. Hete Amp: heterozygous amplification; Hete Del: heterozygous deletion; Homo Amp: homozygous amplification; Homo Del: homozygous deletion; None: no CNV.

To identify the genetic alternations of the GLNM regulators, we assessed the SNV frequency and variant types across 33 types of cancer. The cancer types ranked from high to low by SNV frequency were UCEC (130%), SKCM (61%), COAD (49%), STAD (44%), BLCA (24%), LUAD (24%), LUSC (18%), HNSC (15%), CESC (14%), GBM (12%), KIRP (11%), and BRCA (10%). The SNV frequency was less than 10% in the remaining 15 cancer types. There were no mutations of six GLNM regulators in PCPG and UCS (Figure 3A). The genes ranked from high to low by SNV frequency were *SLC3A2* (27%), *SLC7A11* (23%), *GLS* (20%), *SLC7A5* (18%), *GLS2* (17%), and *SLC1A5* (17%) (Figure 3B). The mutation types included missense mutation, in-frame deletion, nonsense mutation, splice site, frame-shift deletion, frame-shift insertion, multi-hits, and missense mutation, which was the most abundant type. In general, the mutation frequencies of *SLC3A2* (27%) and *SLC7A11* (23%) were the highest and the main mutation type observed was the missense mutation.

We then explored the proportions of the different CNV types of the GLNM regulators, as well as the correlation between the CNV and the mRNA expression. The pie chart shows that the main variation types were heterozygous amplification and heterozygous deletion (Figure 3C). Heterozygous amplifications with percentages >25% were found for *SLC7A11* in ACC and KICH; *SLC7A5* in ACC, KIRP, KICH, and READ; *SLC3A2* in KICH, ESCA, UCS, and OV; *SLC1A5* in ACC, KICH, CESC, GBM, BLCA, LUSC, and UCS; *GLS* in READ, ESCA, LUAD, LUSC, UCS, OV, and TGCT; and *GLS2* in ACC, KIRP, KICH, LUAD, LUSC, UCS, OV, and TGCT. Heterozygous deletions with percentages >25% were found for *SLC7A11* in COAD, READ, HNSC, ESCA, STAD, CESC, CHOL, BLCA, MESO, LUSC, UCS, LIHC, OV, and TGCT; *SLC7A5* in ESCA, LUAD, BLCA, LUSC, UCS, PRAD, SKCM, LIHC, BRCA, OV, SARC, and TGCT; *SLC3A2* in UCS, SKCM and TGCT, *SLC1A5* in LUAD, LGG, OV, SARC, and TGCT; and *GLS* in KICH, BLCA, and SARC. The correlation analysis indicated that the mRNA expression of these regulators was significantly positively correlated with the CNV in the majority of cancer types (*p* < 0.05, Figure 3D). These results suggested that heterozygous amplification and heterozygous deletion were common among these regulators, mediating their abnormal expression and possibly playing an essential role in cancer development.

### 3.3. Epigenetic Alteration of GLNM Regulators in Various Cancers

**Figure 4:**
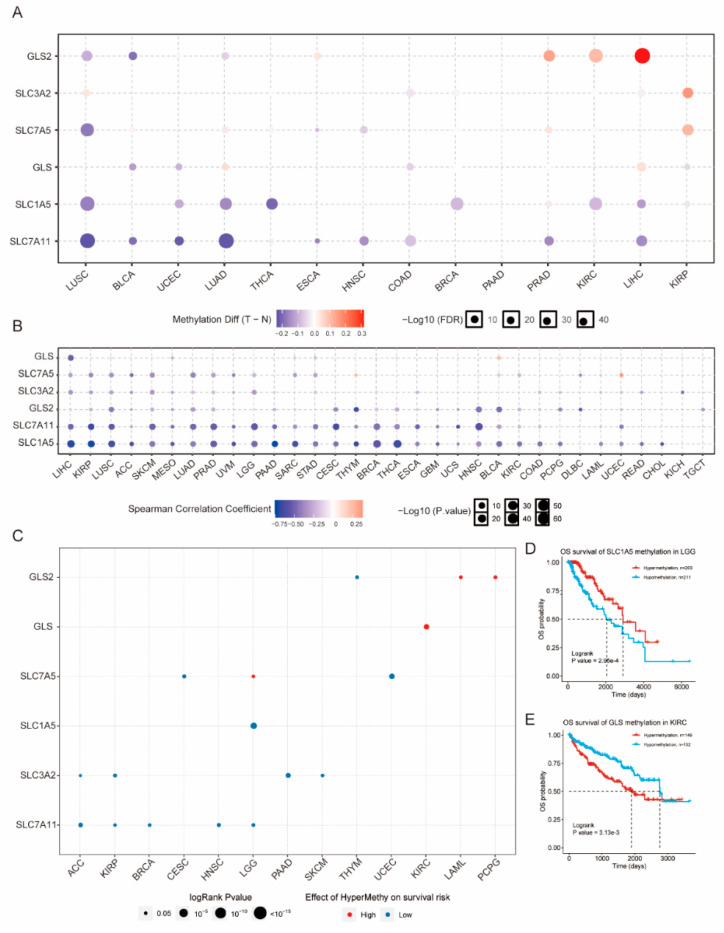
Alterations of glutamine metabolism (GLNM) regulators in terms of methylation in relation to gene expression and overall survival (OS): (**A**) Differential methylation of GLNM regulators between 14 paired normal and tumor tissues; (**B**) Correlation between mRNA expression and methylation. (**C**) Effects of hypermethylation of GLNM regulators on survival risk. Blue dots represent low risk and red dots represent high risk. The sizes of the dots represent significance. (**D**,**E**) KM plots showing the differences in overall survival between patients in low and high hypermethylation groups for *SLC1A5* in LGG (**D**) and *GLS* in KIRC (**E**).

Differential methylation analysis showed that the methylation levels were significantly lower than those of normal tissues for the following: *SLC7A11* in LUSC, BLCA, UCEC, LUAD, ESCA, HNSC, COAD, PRAD, and LIHC; *SLC1A5* in LUSC, UCEC, LUAD, THCA, BRCA, KIRC, and LIHC; *GLS* in BLCA, UCEC, COAD, and KIRP; *SLC7A5* in LUSC, ESCA, and HNSC; *SLC3A2* in COAD; and *GLS2* in LUSC, BLCA, and LUAD; however, the methylation levels were significantly higher in tumor tissues compared to normal tissues for *GLS* in LUAD and LIHC; *SLC7A5* in PRAD and KIRP; *SLC3A2* in LUSC and KIRP; and *GLS2* in ESCA, PRAD, KIRC, and LIHC (Figure 4A). The analysis of the correlation between methylation and mRNA expression indicated that the methylation of the GLNM regulators was significantly negatively correlated with their expression, except for *GLS* in BLCA and for *SLC7A5* in THYM and UCEC (Figure 4B). The overall survival rates in the hypermethylation and the hypomethylation groups showed that the hypermethylation of *SLC1A5*, *SLC3A2*, and *SLC7A11* was a low-risk factor for survival in most of the cancer types; however, hypermethylation was identified as a high-risk factor for survival for *SLC7A5* in LGG, *GLS* in KIRC, and *GLS2* in LAML and PCPG (Figure 4C,D). These results indicated that the abnormal expression of the GLNM regulators could be regulated by aberrant DNA methylation, which could ultimately influence the prognosis of cancer patients.

### 3.4. Significant Correlation of GLNM Regulators with Survival

**Figure 5:**
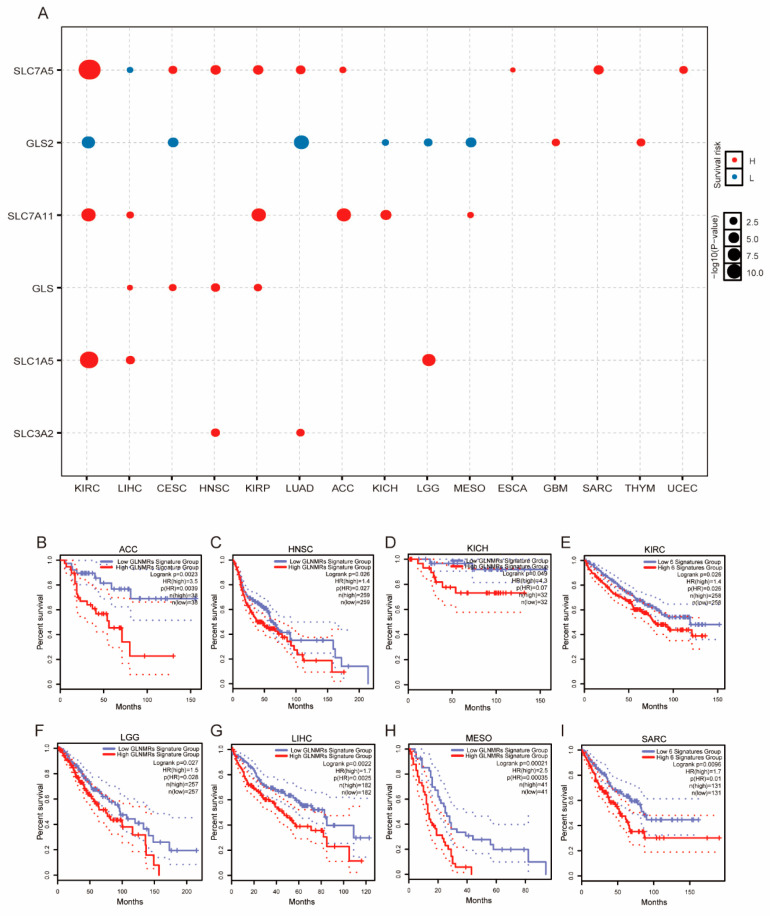
Glutamine metabolism (GLNM) regulator expression associated with prognosis in pan-cancer: (**A**) Heatmap displaying the −log10(*p*-value) of univariate Cox proportional hazards regression between GLNM regulator expression and overall survival (OS). Blue dots represent low-risk genes and red dots represent high-risk genes. The sizes of the dots represent significance. (**B**–**I**) KM plot showing the differences in overall survival between patients in low- and high-GLNMR signature group for (**B**) ACC, (**C**) HNSC, (**D**) KICH, (**E**) KIRC, (**F**) LGG, (**G**) LIHC, (**H**) MESO and (**I**) SARC (all *p* < 0.05). Significance indicated by log-rank tests.

The survival analysis showed that patients with high expression levels of *SLC7A5*, *SLC7A11*, *GLS*, *SLC1A5*, and *SLC3A2* had significantly poorer prognoses for most of the cancer types, while those with high expression of *GLS2* exhibited significantly better prognoses for KIRC, CESC, LUAD, KICH, LGG, and MESO (Figure 5A). To further assess the integrated effects of the GLNM regulators on survival, we performed a survival analysis through the GEPIA2 platform based on a six-gene signature (the GLNMR signature). The results showed that patients in the low-GLNMR signature group had a better overall survival rate than those in the high-GLNMR group in terms of ACC, HNSC, KICH, KIRC, LGG, LIHC, MESO, and SARC (all *p* < 0.05, Figure 5B–I); however, there were no significant differences between the two groups in terms of CESC, ESCA, GBM, KIRP, LUAD, THYM, and UCEC (Figure S1). These results demonstrate that the dysregulated expression patterns of the GLNM regulators were closely associated with the prognoses of patients with ACC, HNSC, KICH, KIRC, LGG, LIHC, MESO, and SARC.

### 3.5. Association of Immune Cell Infiltration and PD-L1 with GLNM Regulators

**Figure 6:**
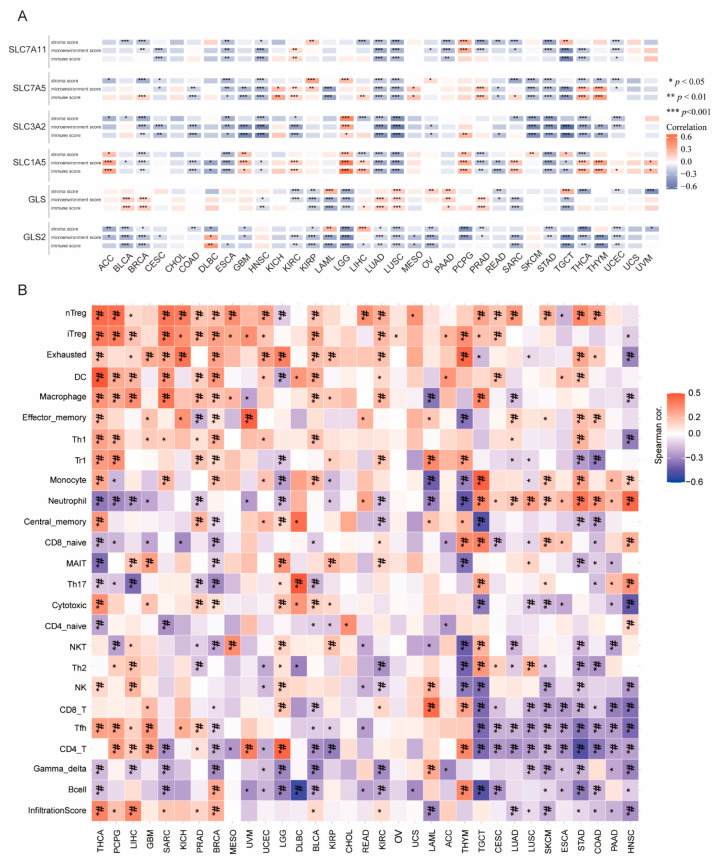
Associations between glutamine metabolism (GLNM) regulator expression and the tumor microenvironment: (**A**) heatmap displaying the correlations between GLNM regulator expression and immune scores, stroma scores, and microenvironment scores; (**B**) the relationship between GLNM regulators expression and immune cell infiltration. Blue represents negative correlations and red represents positive correlations. Note: * *p* < 0.05, ** *p* < 0.01, *** *p* < 0.001, # FDR < 0.05.

**Figure 7:**
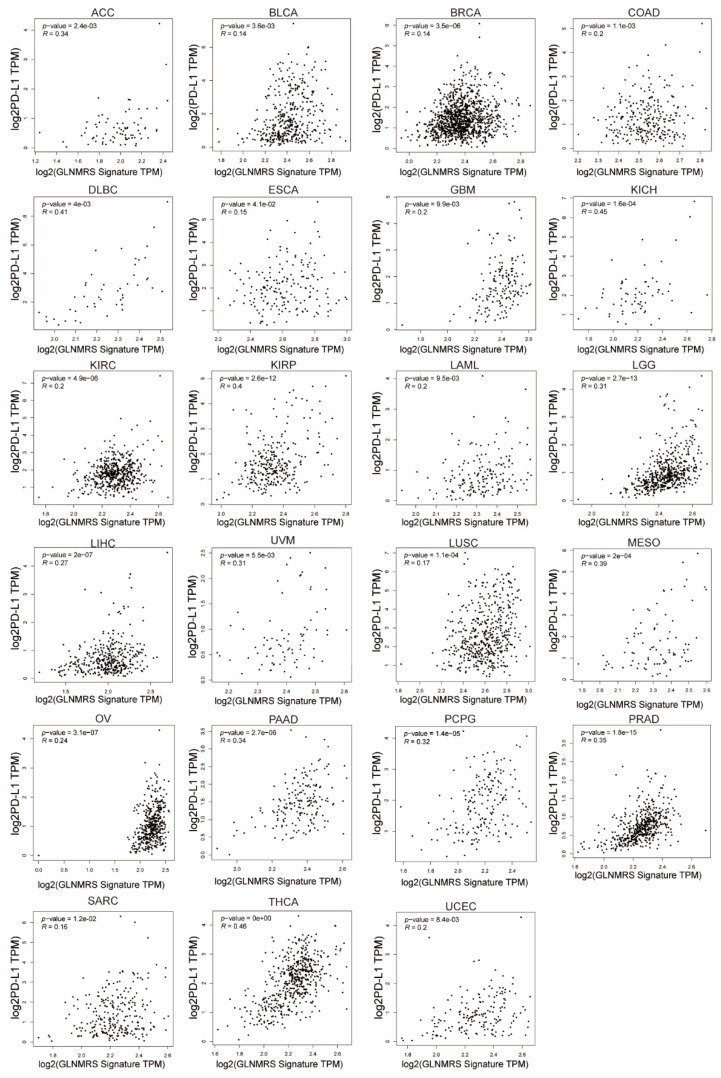
Glutamine metabolism (GLNM) regulator expression is significantly positive correlated with PD-L1 expression in ACC, BLCA, BRCA, COAD, DLBC, ESCA, GBM, KICH, KIRC, KIRP, LAML, LGG, LIHC, LUSC, MESO, OV, PAAD, PCPG, PRAD, SARC, THCA, UCEC, and UVM (all *p* < 0.05).

To explore the involvement of glutamine metabolism in TME, we assessed the correlation of immune cell infiltration and PD-L1 with the GLNM regulators. We found that the immune score, stroma score, and microenvironment score were significantly related to the expression of the GLNM regulators (Figure 6A). There were significantly negative relationships between the immune scores and the GLNM regulators for ESCA, HNSC, KIRP, LAML, OV, READ, SKCM, STAD, TGCT, and UCEC. Next, we further assessed the correlations between the infiltration of 24 immune cells and gene set expression scores (GSVA scores). Based on the correlation patterns between the immune cell infiltrations and the GSVA scores of GLNM regulators, we observed two clusters of cancer types (Figure 6B). These two clusters displayed distinct patterns of immune cell infiltration. Significantly positive correlations were found between the expression of GLNM regulators and the infiltration of immunosuppressive cells (including nTreg, iTreg, exhausted, DC and macrophage cells) in THCA, PCPG, LIHC, GBM, SARC, KICH, PRAD, BRCA, MESO, and UVM. Significantly negative associations were found between the expression of GLNM regulators and the infiltration of the following immune effector cells: NK, CD8+ T, Tfh and CD4+ T cells in TGCT, CESC, LUAD, LUSC, SKCM, ESCA, STAD, COAD, PAAD, and HNSC. We then explored the relationship between the expression level of PD-L1 and the GLNM regulators. We observed that the expression of PD-L1 had significantly positive associations with the expression of GLNM regulators in 23 types of cancers, including ACC, BLCA, BRCA, COAD, DLBC, ESCA, GBM, KICH, KIRC, KIRP, LAML, LGG, LIHC, LUSC, MESO, OV, PAAD, PCPG, PRAD, SARC, THCA, UCEC, and UVM (Figure 7); however, there were no significant associations between PD-L1 expression and the expression of GLNM regulators in the remaining ten cancer types (Figure S2). In general, we demonstrated that increased expression of GLNM regulators was associated with a cold tumor immune microenvironment in almost all cancer types and upregulated PD-L1 expression in ACC, KICH, KIRC, LGG, LIHC, MESO, and SARC, which implied a positive association with immunotherapeutic responsiveness.

### 3.6. Effects of Aberrant GLNM Regulator Expression on Drug Sensitivity

**Figure 8:**
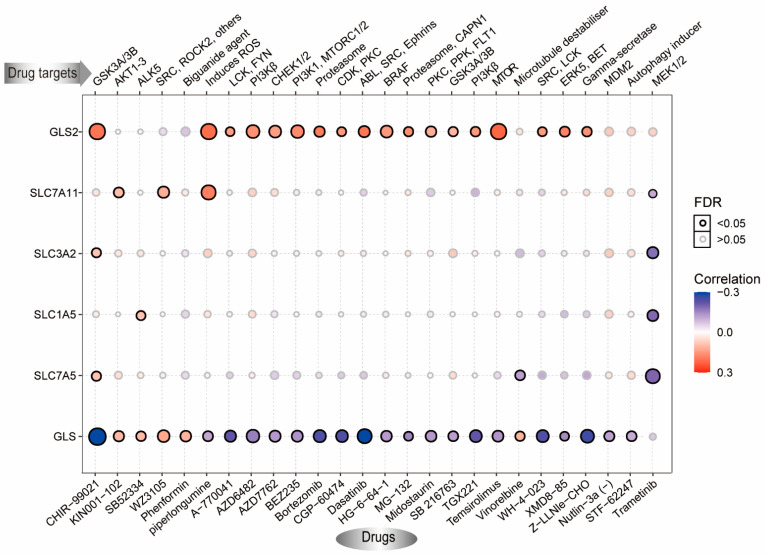
Effects of glutamine metabolism (GLNM) regulator expression on drug sensitivity based on the GDSC database. The horizontal axis represents different drugs, with the paired drug targets displayed above. Blue represents negative correlations and red represents positive correlations. Black circles indicate FDR < 0.05 and gray circles indicate FDR > 0.05.

To elucidate the influence of the GLNM regulators on the effects of drug treatment, we used the GDSC data to investigate the relationship between gene expression and drug sensitivity. We found that *GLS2* expression was positively correlated with resistance to CHIR-99021, piperlongumine, A-770041, AZD6482, AZD7762, BEZ235, bortezomib, CGP-60474, dasatinib, HG-6-64-1, MG-132, midostaurin, SB216763, TGX221, temsirolimus, WH-4-023, XMD8-85, and Z-LLNle-CHO. In contrast, *GLS* expression was negatively associated with the resistance to the drugs stated above. The expression of *SLC7A11*, *SLC3A2*, and *SLC1A5* showed a significantly positive correlation with drug resistance (Figure 8). These results indicated that changes in the expression of GLNM regulators may be an effective indicator for predicting drug responses and could serve as a potential treatment target.

### 3.7. Underlying Molecular Mechanism of GLNM Regulator Alteration

**Figure 9:**
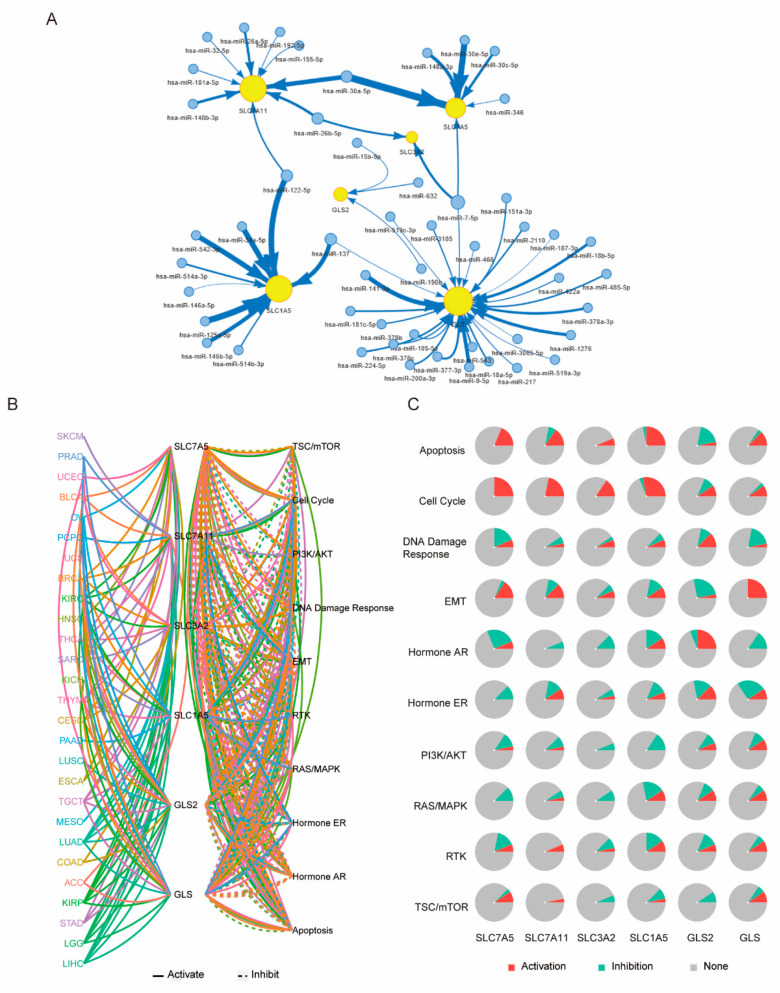
Underlying molecular mechanism of glutamine metabolism (GLNM) regulator expression alterations: (**A**) The miRNA regulation network. Nodes represents miRNAs or target genes, edges represent regulation of miRNA-to-gene transition, and edge widths represent absolute correlation coefficient values. (**B**) A network displaying the relationship between genes and pathways via line connections. Solid lines indicate activation, dashed lines indicate inhibition. Line colors represent different cancer types. (**C**) Pie plots showing the global percentages of cancers in which a gene has an effect on the pathways among 32 cancers types.

The miRNA-to-gene network analysis showed that all six of the GLNM regulators were regulated by more than one miRNA. *SLC3A2* is regulated by only two miRNAs, while *GLS* is regulated by 27 miRNAs. In addition, the same miRNA could regulate multiple genes, such as hsa-miR-7-5p, which regulated *SLC3A2*, *SLC7A5*, and *GLS* (Figure 9A). This indicated that a complex miRNA regulatory network finely regulated the expression of the target GLNM regulators and was involved in tumor development and progression. The pathway analysis showed that the GLNM regulators were involved in TSC/mTOR, the cell cycle, PI3K/AKT, the DNA damage response, EMT, RTK, RAS/MAPK, hormone ER, hormone AR, and apoptosis pathways. The percentage of cancers in which a GLNM regulator had an effect on a certain pathway showed that the transporters, including *SLC7A5*, *SLC7A11*, *SLC3A2*, and *SLC1A5*, were involved in the activation of apoptosis and the cell cycle in most cancer types, while *GLS2* was mainly involved in the activation of hormone AR and *GLS* was mainly involved in the activation of EMT (Figure 9B). These data suggested that the GLNM regulators might play vital roles in regulating cancer-related pathways.

## 4. Discussion

Proper expression of the GLNM regulators is essential for maintaining the balance between glutamine metabolism and cell survival. In the present study, we systemically investigated the relationships between the expression of GLNM regulators and genomic alterations, TME characteristics, prognosis, drug sensitivity, and the underlying molecular mechanisms. Our study demonstrated that in most cancer types, *SLC1A5*, *SLC7A5*, *SLC7A11*, and *SLC3A2* were highly expressed and indicative of poorer survival outcomes, while the effects of changes in the expression of *GLS2* and *GLS* depended on the type of cancer under consideration. High *GLS2* expression was associated with a better prognosis, while high *GLS* expression was associated with a poorer prognosis. In previous studies, the glutamine transporters *SLC1A5*, *SLC7A5*, *SLC7A11*, and *SLC3A2* were found to be highly expressed in a variety of cancers [27,28,29,30]. These transporters mediate glutamine transport, playing a vital role in tumor cell metabolism, proliferation, and cancer prognosis. *GLS* and *GLS2* appeared to exhibit different expression patterns and functions in different types of tumors. In addition, the two glutaminases exhibited diverse roles in tumorigenesis and were shown to suppress or promote tumor development, depending on the specific tumor type. *GLS2* expression was scarce in hepatocellular carcinomas and glioblastomas, which showed high levels of *GLS* [31]. Based on cell line analysis, it was found that the expression of *GLS2* was inversely correlated with *GLS* in 52% of the 33 cancer types. Moreover, it has been reported that *GLS* is frequently upregulated in most cancers, which is somewhat different from our results [31,32]. In the present study, *GLS2* expression and overall survival were positively correlated in most of cancers, with *GLS2* presenting as a suppressor gene. The normal tissue samples in the TCGA dataset were taken from tissues near tumors and may be similar to, but not representative of, true normal tissue. The comparison of tumor tissues with relatively distant normal tissues might have allowed for a more accurate evaluation of differentially expressed genes. This issue may have been responsible for some of the contradictions between the identified upregulation of GLNM regulators in previous studies and our own observation of no significant upregulation in certain cancer types. In general, these regulators could serve as good predictors of the prognosis of different cancer types.

Our results reveal that complex genomic and epigenomic modulations of the GLNM regulators affected their expression, resulting in the metabolic reprogramming of cancer. A previous study had demonstrated that the epigenetic silencing of *GLS2* by promoter hypermethylation was common in human liver cancer, in accordance with the results observed in our study [33]. Additionally, the upregulation of GLNM regulators through miRNAs appears to be another important mechanism. For example, miR-122 has been reported to modulate the expression of *SLC1A5* in hepatocellular carcinoma [34]. In addition, miR-26b has been shown to target *SLC7A11* [17]. We confirmed some of the results shown in previous studies, further supporting the reliability of our findings. We also identified new miRNAs that had not been previously studied. These results can provide theoretical and experimental evidence of the development of safe and effective tumor-targeted drugs. Mechanically, these GLNM regulators are tightly correlated with the activation and inhibition of cancer-related pathways. It was demonstrated that in multiple cancer types, higher *GLS2* expression was negatively correlated with an EMT signature, which may shed light on the mechanism of tumor suppression by *GLS2*. *GLS2* has been identified as a p53 target gene, which may contribute to its tumor suppressor function [35]. In the liver orthotopic model, intrahepatic metastasis and lung metastasis were inhibited by overexpression of *GLS2* [36]. Knockdown of endogenous *GLS2* by shRNA promoted lung metastasis of liver cells in nude mice [37] Mechanistically, *GLS2* negatively regulates the activity of the PI3K-AKT signaling pathway and Rac1 by mediating p53 function [37]. Although *GLS2* tends to exhibit tumor-suppressing activity, it was reported as a pro-tumorigenic gene in luminal-subtype breast tumors [38]. Actually, the expression of *GLS2* is also regulated by oncoproteins including N-myc; therefore, *GLS2* may display complex and diverse modulatory functions in cancer cells. These regulators are functionally coupled, and the whole dynamic process of glutamine metabolism cannot be successfully evaluated in terms of single regulators; thus, we comprehensively evaluated the differences in gene expression between tumors and normal tissues, as well as the prognostic differences between the high-expression and low-expression groups. Comprehensive gene expression profiles can better reflect dynamic metabolic processes, such as glutamine uptake and glutaminolysis.

Although there has been a rapid increase in immunotherapy, the limited beneficiary population and drug resistance are hindering its further development. Metabolically reprogrammed tumor cells compete with immune cells for nutrients and release metabolites into the microenvironment, which inhibit the function of antitumor immune cells; thus, blocking the abnormal metabolism of tumor cells may be a potential way to improve the efficacy of immunotherapy. Previous studies have reported that in breast cancer, glutamine metabolism alterations increased the secretion of G-CSF and GM-CSF, then recruited MDSC to promote tumor progression [39]. The enhanced glutamine uptake influenced the composition of the immune cell infiltrates and was significantly associated with upregulated PDL1 expression as well as poor survival outcomes in breast cancer [40]. In this study, we found that the alteration of the GLNM regulators led to a cold TME, which was negatively correlated with the immune score and immunoeffector cells and positively correlated with immunosuppressive cells. Interestingly, in different tumors, the cold immune microenvironment had distinct phenotypes. Some tumors presented with substantial immunosuppressive cell infiltration, while others presented with decreased infiltration of immunoeffector cells. In addition, the high expression of the GLNM regulators was significantly positively correlated with PD-L1 expression, as was the case in previous studies. Previous studies have demonstrated that the expression of PD-L1 was upregulated in renal cancer cells in glutamine deprivation culture medium via the EGFR/ERK/C-Jun pathway [41]. The expression of PD-L1 was mediated via the nuclear factor-kappa B (NF-kB) signaling pathway, which was activated by the reduction in GSH levels [42]. Upregulated *SLC7A11* promoted the expression of PD-L1 and CSF1 through the αKG/HIF1α axis, which further recruited TAMs and MDSCs infiltration [43]. These results suggested that targeting these regulators may provide a way in which to reverse the cold immune microenvironment and enhance the antitumor effects. For tumors with high expression levels of the regulators and PD-L1, a combination of targeted therapy and immunotherapy may yield better results. Our analysis is the first to identify the two distinct TME patterns affected by the alterations of GLNM regulators across 33 cancer types; however, the underlying mechanism of the effects of these GLNM regulators on the immune microenvironment requires further research.

Due to the rapid proliferation of tumor cells, there is an increased need for glutamine and the GLNM regulators are frequently overexpressed to meet this demand. Regulators that specifically maintained this biological process in tumor cells can be used to develop a novel class of anticancer drugs; the logical basis for this strategy is depriving tumor cells of an important nutrient. Several inhibitors and blockers have been proposed to selectively target the regulators that are abnormally expressed in cancer [8]; however, all of these agents remain in the preclinical stage. *SLC7A11* has been reported to induce selective drug resistance [15,16,17,44]. Recently, *SLC7A11* was also reported to be involved in the sensitivity to the histone deacetylase inhibitors [45]; thus, the pharmacological blockade of *SLC7A11* not only inhibits tumor growth, but also reverses resistance to certain drugs. In the present study, we observed that the high expression of *GLS* was positively or negatively correlated with resistance to specific drugs. We found that *GLS* expression was negatively associated with the sensitivity of temsirolimus, an mTOR inhibitor. Interestingly, combining the *GLS* inhibitor with an mTOR inhibitor is reported to result in a combinatorial effect [46,47,48]. The upregulation of *GLS* was reported to be induced following mTOR inhibition, providing a theoretical basis for the combination of *GLS* inhibitors and mTOR inhibitors. Moreover, *GLS* was identified as an activator of the mTOR pathway to promote colorectal carcinogenesis; therefore, we speculated that the increased expression of *GLS* would activate the mTOR signaling pathway, as the expression of *GLS* was negatively correlated with the sensitivity of mTOR inhibitors. On the other hand, we speculated that the use of mTOR inhibitors would upregulate the expression of *GLS* through a feedback mechanism, leading to drug resistance of mTOR inhibitors, meaning combined use would increase the efficacy. These correlations warrant further in-depth studies in a certain type of tumors. The relationship of GLNM regulator expression and drug sensitivity might provide an important theoretical basis for developing novel cancer treatments. Additionally, these findings provide a rationale for combination therapies to reverse resistance to certain drugs [49].

Despite the new insights into the alternations of GLNM regulators provided by our study, we note that there are some limitations. First, based on information from the TCGA, we could only assess the genetic changes in the tumor tissue as a whole, and it was not feasible for us to analyze the genetic changes in tumor cells and stromal cells separately. Second, we explored only a few potential alterations that can affect the expression and function of the GLNM regulators. Other related alterations that should be considered are post-transcriptional and post-translational modifications, such as mRNA splicing, m6A methylation, and altered protein stability. Third, since our study was mainly based on gene expression, we could not gain insights into the impacts of the CNV and SNV alternations on tumors. Finally, we did not perform any analyses that correlated the expression levels of GLNM regulators with those of demographic factors or pathologic factors other than overall survival.

## 5. Conclusions

In this study, we focused on the expression and function of six key GLNM regulators in tumors and the TME. Exploring the genomic alterations and miRNA network revealed additional mechanisms of the cancer-associated dysregulation of these regulators. Our results were in accordance with those of most previous studies and provided new information for subsequent studies. In addition, the significant effects of these regulators on the TME and drug resistance were identified, which provides a novel insight into cancer treatment and may offer alternative options for the treatment of clinically refractory cancers.

## Data Availability

The datasets analyzed during the current study are available in The Cancer Genome Atlas (https://portal.gdc.cancer.gov/) and GTEx expression dataset (V7.0).

## Supplementary Materials

The following are available online at https://www.mdpi.com/article/10.3390/genes12091305/s1, Figure S1: KM plot showing the differences in overall survival between patients in low- and high-GLNMR signature group in (A) CESC, (B) ESCA, (C) GBM, (D) KIRP, (E) LUAD, (F) THYM, and (G) UCEC. (All *p* > 0.05). Figure S2: Scatter plot showing relationships between glutamine metabolism (GLNM) regulator expression and PD-L1 expression in CESC, CHOL, HNSC, READ, SKCM, STAD, TGCT, THYM, and UCS. (All *p* > 0.05)
