# Supplementary material for: Glutamine Metabolism Regulators Associated with Cancer Development and the Tumor Microenvironment: A Pan-Cancer Multi-Omics Analysis
Source: Genes (Basel). 2021 Aug 25;12(9):1305. doi: 10.3390/genes12091305 (PMC8466418; doi:10.3390/genes12091305)
Supplement: Supplementary file 1 [file genes-12-01305-s001.zip › genes-1291631-supplementary.pdf]

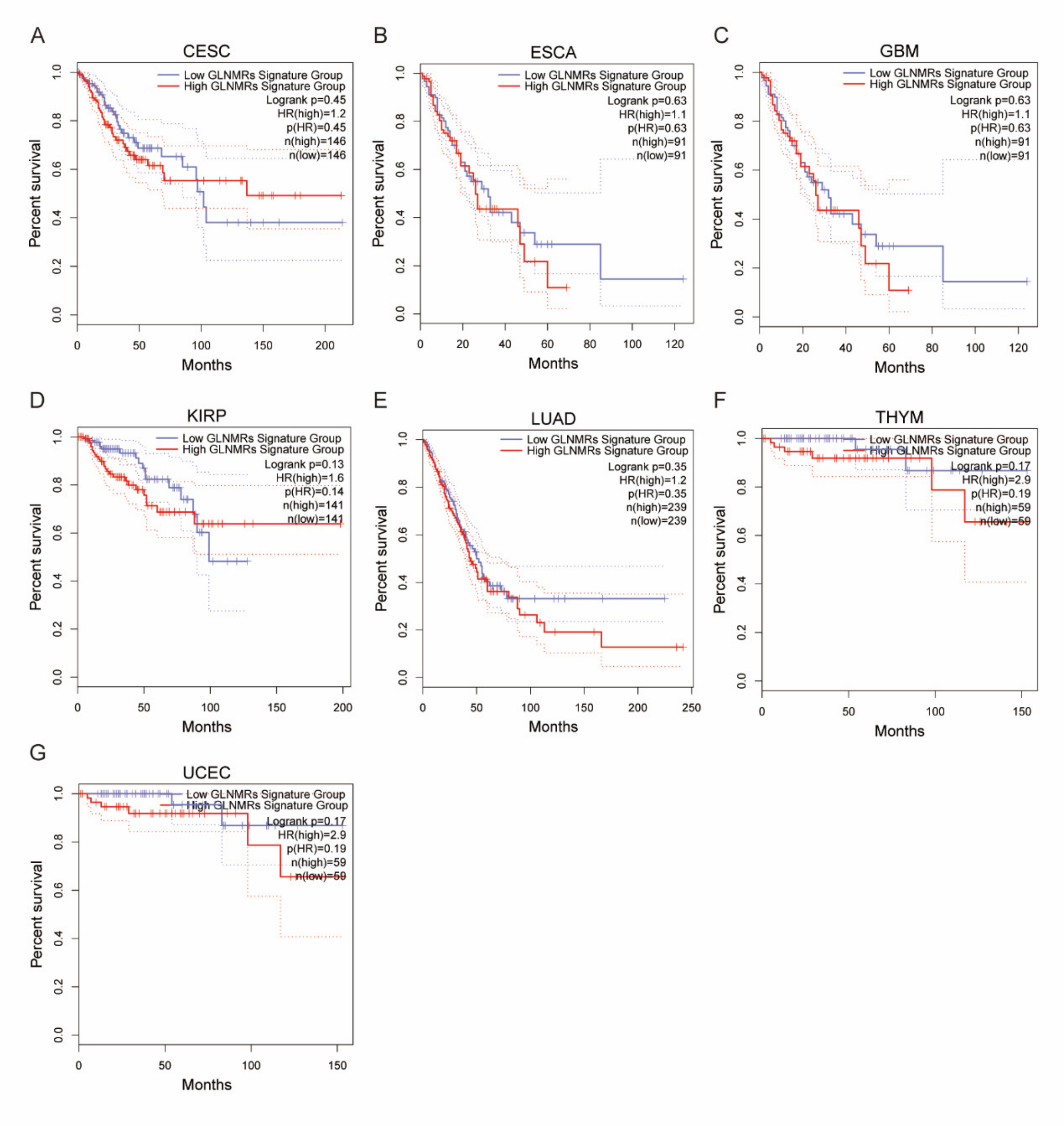

Figure S1. KM plot showing the differences in overall survival between patients in low- and high-GLNMR.signature group in (A) CESC, (B) ESCA, (C) GBM, (D) KIRP, (E) LUAD, (F) THYM, and (G) UCEC.(All p > 0.05).

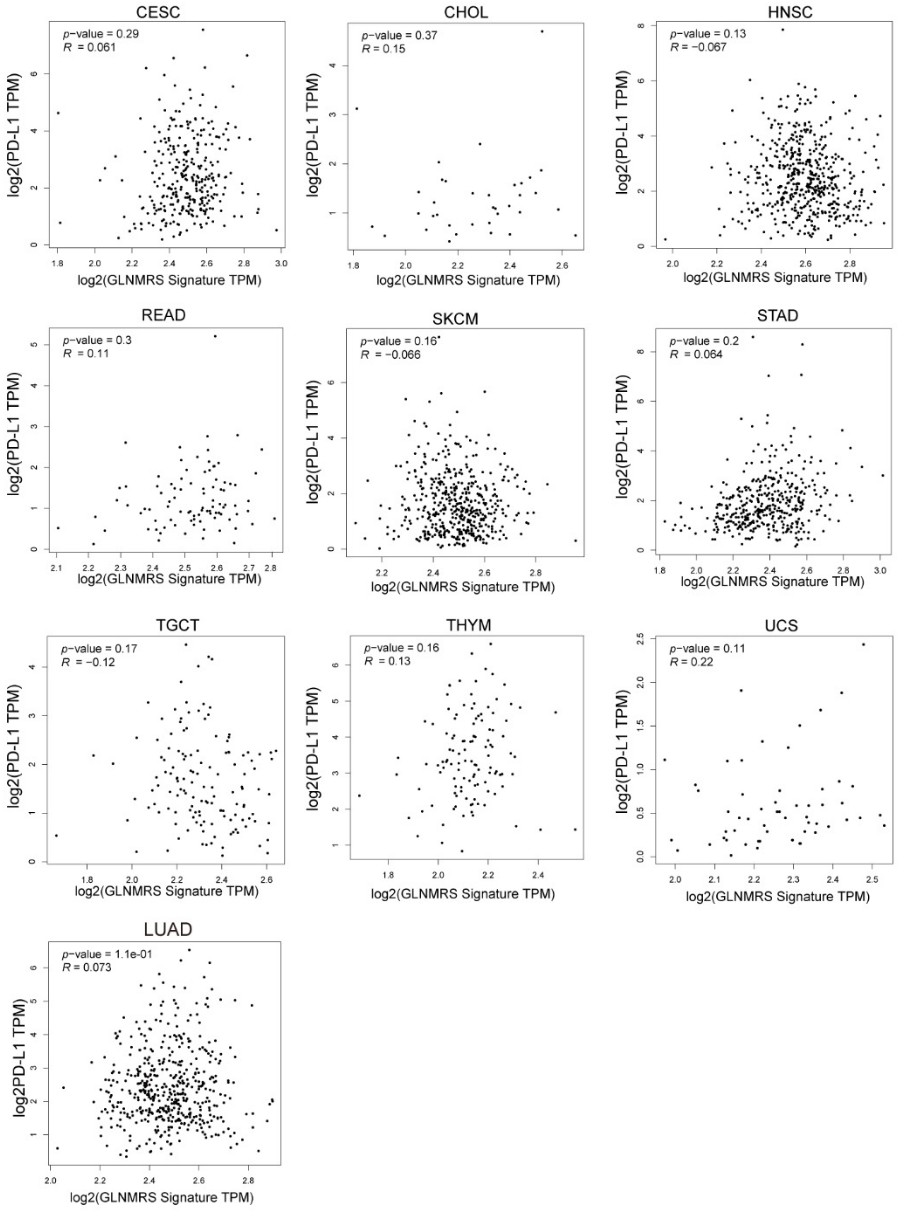

Figure S2. Scatter plot showing relationships between glutamine metabolism (GLNM).regulator expression and PD-L1 expression in CESC, CHOL, HNSC, READ, SKCM, STAD, TGCT,THYM, and UCS. (All p > 0.05)
